# Mindfulness-Based Programs for Patients With Cancer via eHealth and Mobile Health: Systematic Review and Synthesis of Quantitative Research

**DOI:** 10.2196/20709

**Published:** 2020-11-16

**Authors:** Juraj Matis, Miroslav Svetlak, Alena Slezackova, Marek Svoboda, Rastislav Šumec

**Affiliations:** 1 Department of Psychology Faculty of Arts Masaryk University Brno Czech Republic; 2 Department of Psychology and Psychosomatics Faculty of Medicine Masaryk University Brno Czech Republic; 3 Department of Psychiatry Faculty of Medicine Masaryk University Brno Czech Republic; 4 Department of Comprehensive Cancer Care Masaryk Memorial Cancer Institute Brno Czech Republic; 5 First Department of Neurology Faculty of Medicine Masaryk University and St. Anne’s University Hospital Brno Czech Republic

**Keywords:** eHealth, mHealth, mindfulness, cancer, systematic review, mobile phone

## Abstract

**Background:**

eHealth mindfulness-based programs (eMBPs) are on the rise in complex oncology and palliative care. However, we are still at the beginning of answering the questions of how effective eMBPs are and for whom, and what kinds of delivery modes are the most efficient.

**Objective:**

This systematic review aims to examine the feasibility and efficacy of eMBPs in improving the mental health and well-being of patients with cancer, to describe intervention characteristics and delivery modes of these programs, and to summarize the results of the included studies in terms of moderators, mediators, and predictors of efficacy, adherence, and attrition.

**Methods:**

In total, 4 databases (PubMed, PsycINFO, Scopus, and Web of Knowledge) were searched using relevant search terms (eg, mindfulness, program, eHealth, neoplasm) and their variations. No restrictions were imposed on language or publication type. The results of the efficacy of eMBPs were synthesized through the summarizing effect estimates method.

**Results:**

A total of 29 published papers describing 24 original studies were included in this review. In general, the results indicate that eMBPs have the potential to reduce the levels of stress, anxiety, depression, fatigue, sleep problems, and pain, and improve the levels of mindfulness, posttraumatic growth, and some parameters of general health. The largest median of Cohen d effect sizes were observed in reducing anxiety and depression (within-subject: median −0.38, IQR −0.62 to −0.27; between-group: median −0.42, IQR −0.58 to −0.22) and facilitating posttraumatic growth (within-subject: median 0.42, IQR 0.35 to 0.48; between-group: median 0.32, IQR 0.22 to 0.39). The efficacy of eMBP may be comparable with that of parallel, face-to-face MBPs in some cases. All studies that evaluated the feasibility of eMBPs reported that they are feasible for patients with cancer. Potential moderators, mediators, and predictors of the efficacy, attrition, and adherence of eMBPs are discussed.

**Conclusions:**

Although the effects of the reviewed studies were highly heterogeneous, the review provides evidence that eMBPs are an appropriate way for mindfulness practice to be delivered to patients with cancer. Thus far, existing eMBPs have mostly attempted to convert proven face-to-face mindfulness programs to the eHealth mode. They have not yet fully exploited the potential of eHealth technology.

## Introduction

### Background

Cancer is the second leading cause of death in the world, and approximately 1 in 6 deaths is because of this disease [1]. Owing to medical care, the life of patients has been increasingly prolonged for some types of cancer and associated somatic symptoms such as pain, fatigue, and nausea are better controlled. In this context, health care professionals face the challenge of helping more patients with cancer than ever before to live their lives more fully. Psychosocial distress associated with life for patients with cancer has been identified as a significant problem. In a large group of new patients with cancer (3035 patients), 25.7% scored above the cutoff points for distress, anxiety, and depression [2]. The prevalence of major depressive disorders is approximately 15% in patients with advanced cancer [3], and 30% to 40% of patients in various stages of cancer development report significant psychosocial distress symptoms, such as anxiety, depression, nervousness, and insomnia [4-6]. The management of these psychiatric symptoms, especially of distress related to the cancer diagnosis, is one of the main challenges of complex oncological and palliative care.

Psychotherapy, counseling, and other nonpharmacological methods such as mindfulness-based programs (MBPs) are often not implemented, despite their efficacy in standard oncological and palliative care [7,8]. They are underestimated by patients [9] and physicians [10], and they are often unavailable at the appropriate time.

### MBPs

The *first generation* of MBPs, which have the most robust evidence within the field, are mindfulness-based stress reduction (MBSR) and mindfulness-based cognitive therapy (MBCT). The difference between MBSR and MBCT depends partly on the client group at which the course is aimed. MBSR was originally aimed at people with chronic pain and stress [11]. MBCT was aimed at people with an affective disorder, especially those with recurrent depression [12]. MBCT combines systematic mindfulness training with cognitive behavioral therapy to help people with a history of depression learn vital skills. Both involve a manualized 8-week program of meditation and gentle Hatha yoga training. Participants attend weekly group sessions where they are introduced to formal meditation practices, gentle yoga, and psychosocial education. The program also includes a silent meditation retreat day that falls in the second half of the course. Group members are asked to practice for 45 min per day. Participants are encouraged to keep a diary to describe their practice, reflections, and insights. Formal meditation practices include focused attention on breathing, body scans, and open monitoring of sounds, thoughts, feelings, and bodily sensations. Group sessions generally last for 2.5 hours and focus on group meditation practice and discussion of these practices. MBCT is largely based on MBSR, and many of its parts are the same [13].

A growing number of theoretical studies have attempted to operationalize the mindfulness concept. In their oft-cited review, Bishop et al [14] define mindfulness as follows: “Broadly conceptualized, mindfulness has been described as a kind of nonelaborative, nonjudgmental, present-centered awareness in which each thought, feeling, or sensation that arises in the attentional field is acknowledged and accepted as it is.” They further state that, “in the state of mindfulness, thoughts and feelings are observed as events in mind, without overidentifying with them and without reacting to them in an automatic, habitual pattern of reactivity. This dispassionate state of self-observation is thought to introduce a ‘space’ between one’s perception and response. Thus, mindfulness is thought to enable one to respond to situations more reflectively (as opposed to reflexively).”

From the first published article by Kabat-Zinn [11] on the positive effect of MBSR on reducing pain and symptoms of negative mood in a group of patients with chronic pain, the effectiveness of MBPs on improving mental and physical health has been repeatedly documented in healthy people [15,16] and in people with various psychiatric or somatic conditions [17,18]. Numerous subsequent studies have also offered considerable evidence about the benefits of practicing mindfulness meditation for patients facing different types and stages of cancer [19-22]. Recent systematic reviews and meta-analyses have documented the moderate positive effect of MBP on anxiety and depression symptoms in patients with cancer and survivors of cancer [23]; a small effect on depression and a moderate effect on anxiety [24]; moderate-to-large positive effects on the mental health of patients with breast cancer [25]; a moderate effect on anxiety, stress, fatigue, general mood, and sleep disturbance and a small effect on physical health variables in a mix of cancer diagnoses [26]; a medium effect on anxiety, depression, quality of life, fatigue, stress, and posttraumatic growth [27]; and a small-to-medium effect on health-related quality of life, fatigue, sleep, stress, anxiety, and depression [28].

MBPs have been repeatedly shown to be effective in reducing cancer-related pain [29], supporting psychological well-being in adults with advanced cancer [30], reducing depressive symptoms in patients with breast cancer [31], decreasing fear of cancer recurrence [32], and even maintaining telomere length in survivors of breast cancer [33]. Systematic and comprehensive programs based on MBCT for cancer (MBCT-Ca) [34] and mindfulness-based cancer recovery (MBCR) [35] have also been developed.

### eHealth MBPs

There has been an increasing effort to transfer traditional health care practices to the formats of eHealth and mobile health (mHealth) [36] to provide widely accessible psychological support with minimal economic costs to those who need it.

According to Eysenbach [37], eHealth refers to “health services and information delivered or enhanced through the internet and related technologies. In a broader sense, the term characterizes not only a technical development, but also a state-of-mind, a way of thinking, an attitude, and a commitment for networked, global thinking, to improve health care locally, regionally, and worldwide by using information and communication technology.” Later, the term was broadened to include mHealth, adding mobile phones and apps to the definition [36].

Although the therapist-client relationship and dialog are irreplaceable and are key common factors in psychotherapy beyond the effect [38], web-based interventions offer some advantages, specifically for people who are functioning at good or adequate personality levels. Web-based programs (1) are easily accessible, (2) are anonymous, (3) are available 24/7 to people during the course of their daily life, (4) do not necessarily require the involvement of a therapist educated in mindfulness, (5) are less expensive, and (6) save time [39-41]. The preference for web-based delivery is reflected in the increasing number of mindfulness-based mobile apps; a search identified 560 available apps [42].

Increasing evidence supports the advantages and effectiveness of web-based MBPs [41,43,44]. eHealth MBPs (eMBPs) have been shown to be effective in supporting mental health and reducing symptoms of psychopathology in healthy subjects [43,45] and in patients with depression [46], anxiety [47], tinnitus [48], chronic pain [49], and fibromyalgia [50].

In total, 2 recent systematic reviews have documented that eMBPs are feasible and effective for people with various physical health conditions [51] and that the eMBPs are effective in reducing depression and anxiety in clinical populations [52].

An increasing number of studies have revealed that eMBPs are effective and suitable for patients with cancer [53,54]. However, there has not yet been a systematic review of these studies and their descriptions of the interventions in terms of their characteristics, such as delivery mode and approach.

Owing to the heterogeneity in form and content of the eMBPs used in oncology, it is necessary to clarify what an MBP is and what it is not. MBPs typically include mindfulness training via 3 formal mindfulness meditation practices [8]: body scan, mindful movement, and sitting meditation. It is based on daily home practice with the support of recorded guidance. Throughout the program, participants are encouraged to develop their informal practice by bringing awareness in particular ways to everyday life.

According to a study by Crane et al [8], MBPs primarily include MBSR and MBCT, the *first-generation* MBPs. The length, type, and frequency of mindfulness practice are strictly recommended in these programs.

However, as the field has developed, new adaptations of first-generation MBPs have been developed for particular purposes and populations (eg, MBCT-Ca [55] and MBCR [35]). Various adapted MBPs maintain the structure of the first-generation programs and contain the 3 formal mindfulness meditation practices, but they can vary in length and content (eg, with and without a 1-day retreat).

The development in this field has also brought a few approaches, such as acceptance and commitment therapy (ACT) [56] and dialectical behavioral therapy [57] that share several underpinning theoretical ideas with MBPs and some mindfulness meditation practices. These programs could be called *mindfulness-informed programs* [8]. With ACT, there is some promising evidence that it may improve the quality of life, emotional state, psychological flexibility, and possibly disease self-management in patients with cancer [58,59].

Research on eMBP feasibility and efficacy is still in its initial stages in the oncology field. With the aim of describing the heterogeneity of this field and not missing any potentially feasible and effective eMBPs using the mindfulness approach for patients with cancer, every level of MBP is included in this systematic review: first-generation MBPs, adapted MBPs, and mindfulness-informed programs.

The question of the appropriate length and content for an eMBP to have a positive effect on mental health remains open. MBSR and MBCT programs can be considered as the gold standard in this intervention area with an 8-week duration. Some experimental data have revealed that 4-week mindfulness programs seem to be efficacious for promoting well-being and stress reduction [60]. There is also some consensus among experts in this field [61,62] that a 4-week mindfulness training completion can be considered as a minimum adequate *dose*. The prevalent duration of eMBPs listed in 4 recent systematic reviews [41,51,52,60] is, on average, 8 (SD 1.86) weeks (minimum 3 weeks and maximum 12 weeks). The feasibility and effectiveness of short eMBPs have been demonstrated in patients with cancer undergoing chemotherapy [63], but there are few such programs, and they cannot be considered as systematic mindfulness training.

### Aims of This Study

This study aims to examine the feasibility and efficacy of eMBPs in improving mental health and well-being in patients with cancer, to describe the intervention characteristics and delivery modes of these programs, and to summarize the results of the included studies in terms of the moderators, mediators, and predictors of efficacy, adherence, and attrition. This should serve as a starting point for maximizing effectiveness and adherence and minimizing attrition rates in the construction and development of future eMBPs for patients with cancer.

## Methods

### Reporting Guidelines Used

This review was conducted in accordance with the 2009 PRISMA (Preferred Reporting Items for Systematic Reviews and Meta-Analysis) [64] and with supportive guidance from the Cochrane Handbook for Systematic Reviews of Interventions [65]. The protocol of this review was not preregistered.

### Search Strategy

**Figure 1:**
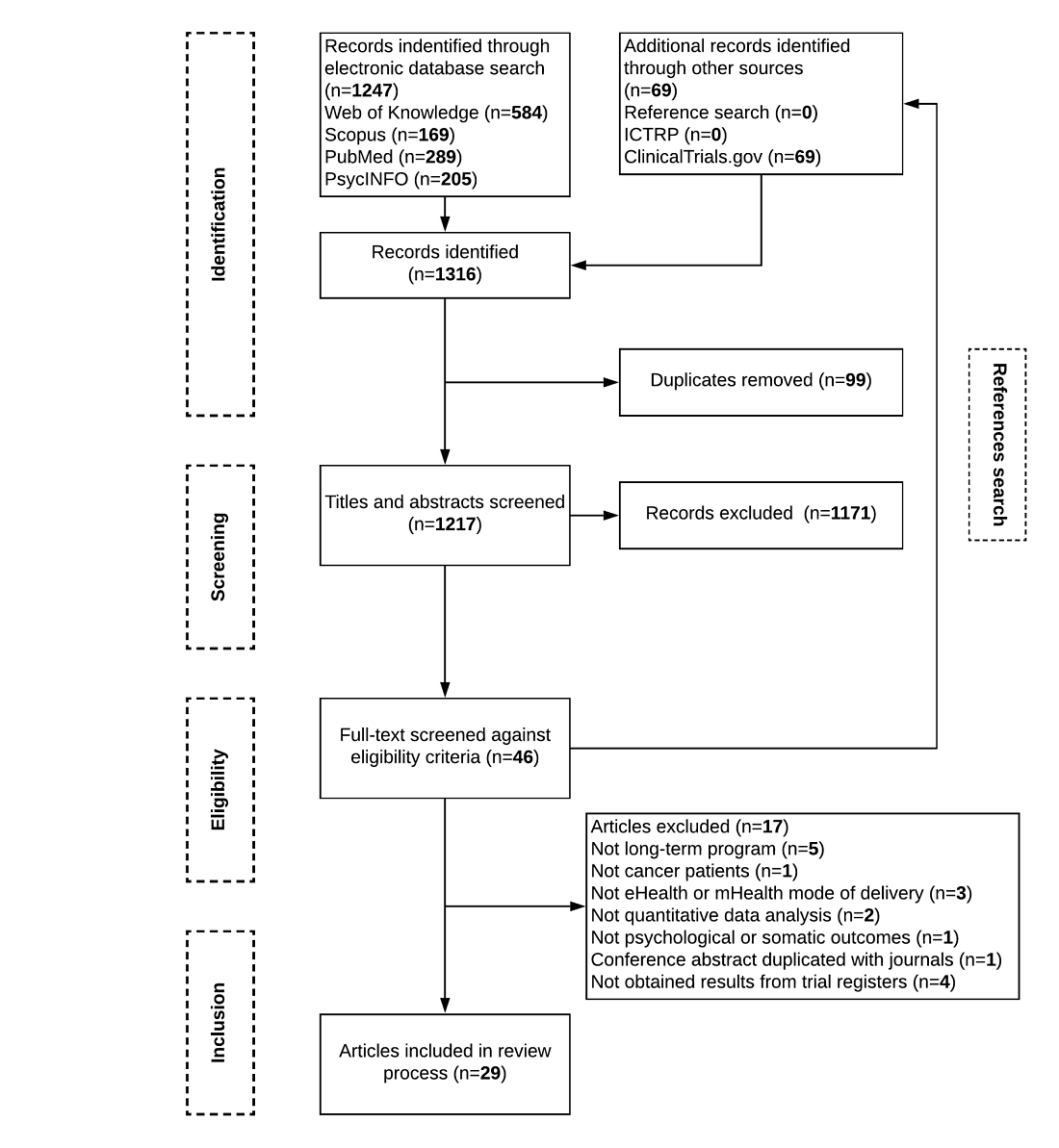
Search and selection process and reasons for exclusion according to the PRISMA (Preferred Reporting Items for Systematic Reviews and Meta-Analysis) guidelines. mHealth: mobile health; ICTRP: International Clinical Trials Registry Platform.

A systematic literature search was conducted in 4 electronic databases: PubMed, Web of Knowledge, Scopus, and PsycINFO. Each database was searched from the first available date until July 31, 2020, using relevant search terms (eg, mindfulness, program, eHealth, and neoplasm) and their variations (all terms used are presented in Multimedia Appendix 1). No restrictions were imposed on language or publication type. The World Health Organization, International Clinical Trials Registry Platform, and the US National Library of Medicine trial registry platform were also searched to detect relevant completed trials that have not yet been published (4 potential trials were found and authors were contacted, but data were not obtained). In addition, the reference lists of the included publications were examined. Figure 1 shows the flowchart of selection and inclusion.

### Selection of Studies

Search terms for the literature search were chosen by 2 authors (JM and MS) and then consulted with the review team. The database search and paper screening (title, abstract, and full text) were undertaken by the same 2 authors (JM and MS); if the eligibility of a study was unclear, the review team discussed it until consensus was reached. One study author was contacted for additional eligibility information; the author responded and supplied the necessary information.

Inclusion criteria were employment of mindfulness in an intervention program, administration of the program via an eHealth mode of delivery (including website, app, videoconference, computer, and telephone), use of quantitative data analysis, evaluation of psychological or somatic outcomes, administration of the program to a patient population with cancer, and program duration of at least 4 weeks.

### Data Extraction

Data extraction was performed by 4 review authors in the first phase (AS, MS, RS, and JM). In the second phase, the extracted data were checked by JM. Disagreements among the review authors were discussed by the review team until consensus was reached. A data extraction sheet was developed and pilot tested on 6 randomly selected included studies and then refined accordingly. Every team member was pretrained in the data extraction process. For each included study, the following data were extracted: first author; country and year of publication; population characteristics, including cancer stage and type, receipt of primary treatment, age, sex (proportion of females in total study population), and number of participants per condition; intervention characteristics, including type (eg, MBSR) and purity (pure mindfulness or combined with other interventions) of intervention, delivery mode (eg, website, telephone), program structure (predefined or nonpredefined; predefined program progress according to guidelines, eg, MBSR; or nonpredefined program progress, eg, free access to program modules based on patient preferences), facilitation (facilitated: synchronous or asynchronous personal contact with the facilitator; nonfacilitated: eMBP without personal facilitation), type and frequency of reminders, presence of a retreat day, number and average time of sessions, and duration of intervention in weeks; comparison group (eg, waitlist, usual care); outcomes and their type (primary or secondary); outcome measurements, duration from baseline to postintervention, or latest available follow-up assessment (eg, 8 weeks); attrition (ie, intervention dropout); adherence to program in terms of session attendance or completion, practice frequency, and practice time or duration; and data to calculate pre-post effect sizes per condition. A total of 3 authors had to be contacted for additional information or missing data; 2 responded and provided the required information or data, and one was not contactable (email out of service).

### Evaluation of Methodological Quality

The methodological quality of randomized controlled trial (RCT) studies was assessed based on potential sources of bias outlined in the Cochrane Handbook for Systematic Reviews of Intervention [65]. In pre-post studies, only those items of the Cochrane assessment tool that fit were used. The presence of a control group item was added because almost half of all included studies did not have a control group. Sources of bias that were assessed in all studies (pre-post studies without control group included) were (1) complete outcome data or intention-to-treat (ITT) analysis used (where the threshold for acceptable dropout rate was determined as ≤15%; note that the 10% attrition cutoff recommended by the Cochrane risk of bias tool [66] was modified and set at 15% in this review as a mean attrition rate of eHealth interventions recently systematically reviewed [51]. This cutoff better reflects the higher attrition rate in the eHealth interventions (and it is slightly less conservative), (2) all outcomes reported, and (3) the presence of a control group. Sources of bias that were also assessed only in RCT studies were random sequence generation, allocation concealment, blinding of participants and personnel, blinding of outcome assessment (note that because of the nature of the included studies, blinding of participants, personnel, and outcome assessment was not possible), and similar groups (ie, whether the groups were similar on prognostic indicators at baseline or appropriate adjustments were made to correct for baseline imbalance). Note that studies were coded as *yes* when they met the criteria, *no* when they did not meet the criteria, and *unclear* when it was ambiguous as to whether they met the criteria. This results in a low, high, and unclear risk of bias, respectively.

Assessment of methodological quality was undertaken by 4 review authors in the first phase (AS, MS, RS, and JM), and data were checked by JM in the second phase. Disagreements among the authors were discussed by the review team until consensus was reached.

### Data Analysis

The adherence rate was computed as the reported amount of practice or sessions (completed by a certain proportion of participants) divided by the intended or recommended amount of the intervention protocol where it was possible. The attrition rate (intervention dropout) was computed as participants who completed the intervention (continued in intervention to the end, regardless of adherence or completion of postintervention assessments) divided by participants enrolled in the intervention. The effect sizes of studies were reported only at the time points when statistical analysis was conducted. When the effect sizes were not reported by the authors and data for the computation were available, they were computed using Cohen *d* formula [67]. For within-subject results, the formula was as follows: pre-post mean change divided by the pooled SDs. For between-group results, the formula was as follows: the difference between groups pre-post mean changes divided by the pooled baseline SDs.

The efficacy of eMBPs was synthesized using the summarizing effect estimates method. This approach was used because the investigation of eMBPs in patients with cancer is still in its early stages (predominantly in stage 1 [68] according to the National Institutes of Health Stage Model [69], as nearly half of the programs in this review included intervention generation, refinement, modification, adaptation, and pilot testing), whereas efficacy results are reported only in a preliminary manner. Thus, there is a small number of RCT studies per outcome and generally high heterogeneity across all included studies—study characteristics (design, intervention type, delivery modes, measured outcomes, and assessment timepoints) varied substantially, and homogeneity in effects cannot be expected with standard meta-analytical methods. With the aim of identifying potential predictors, moderators, and mediators of eMBP attrition, adherence, and efficacy in the reviewed studies, the results of their analysis are summarized within this review. Possible publication biases could not be estimated because of the limited number of studies per outcome.

## Results

### Study Selection

The database search and the search of trial registers provided a total of 1316 results. After removing duplicates and screening titles, abstracts, and full texts against the inclusion criteria, conducting a complementary hand search of the reference lists of eligible studies, and contacting study authors, a total of 29 published papers describing 24 original studies were included in this review. Of the included studies, 5 studies described long-term follow-up results, predictors, and associations or other health-related outcomes that were separately published [53,70-73]. Figure 1 illustrates the search and selection process and reasons for exclusion according to the PRISMA guidelines [64].

### Population Characteristics

The population characteristics of the included studies are reported in Multimedia Appendix 2 [74]. Most of the studies examined mixed cancer types (n=11), mixed staged patients (n=11), included patients after the completion of primary cancer treatment (n=11), and were conducted in the United States (n=15). Cancer stages ranged from 0 to 4. Note that if patients received hormonal therapy only, we considered them as having completed primary cancer treatment.

### Study Characteristics

The study characteristics of the included studies are reported in Multimedia Appendix 2. A total of 2522 adults participated in the study. Of the included studies, 10 were pre-post pilot feasibility studies without an active or waitlist control group [54,75-83] and 14 employed an RCT design [62,71,84-95]. Of those RCTs, 9 studies had a no-intervention control (waitlist usual care group) [62,71,87-89,91,93-95]; two of those 9 studies used additional active specific intervention–face-to-face MBCT program [62] and supportive-expressive group [89]; 4 studies compared eMBP with minimal intervention control (education or enhanced usual care) [84-86,90], one of those 4 studies used an additional specific active intervention–physiotherapist-guided ambulant activity feedback (AAF) therapy encompassing the use of an accelerometer [84], and 1 study compared eMBP with a parallel mindfulness program only [92]. Of the included studies, 10 studies reported follow-up results, with the duration from the postintervention assessment ranging from 4 weeks to 9 months. 

### Intervention Characteristics

The intervention characteristics of the included studies are reported in Multimedia Appendix 2. The primary delivery platform, secondary intervention delivery channel, and type of reminder are summarized in Table 1. The primary platform is an internet or internet-related technology that delivers eMBP. The secondary intervention delivery channel is the concrete way in which the program is delivered to patients. The type of reminder includes the ways in which the patients are reminded of the program in an attempt to increase the patient’s completion rate and adherence to the intervention. The most frequently used primary platforms are websites (n=9) and smartphone apps (n=8). All studies except one [71] used audio recordings as a secondary delivery mode (n=23), usually combined with other channels. Reminders were used in almost half of the studies (n=11), and almost half of them used email (n=5). Of all 24 studies, only 2 used the exact same combination of delivery modes; these 2 studies were conducted by the same research group [77,84]. These results reveal considerable heterogeneity in the primary and secondary delivery modes of the included studies.

**Table 1:** Primary delivery platforms, secondary intervention delivery channels and reminders of eHealth mindfulness-based program of included studies.

Platform		Value, n (%)
**Delivery platforms**		
	Website	9 (38)
	Mobile app	8 (33)
	Videoconference	5 (21)
	Telephone call	3 (13)
	Email	2 (8)
**Delivery channels**		
	Audiorecording	23 (96)
	Video	10 (42)
	Workbook	8 (33)
	Email	2 (8)
**Reminders**		
	Email	5 (45)
	Telephone call	2 (18)
	Text message	1 (9)
	Postcard	1 (9)
	Notification	1 (9)

The eMBPs could be classified according to program structure (predefined program progress according to guidelines, eg, MBSR; nonpredefined program progress, eg, free access to program modules based on patient preferences) and facilitation (facilitated: synchronous or asynchronous personal contact with the facilitator; nonfacilitated: standardized eMBP without personal facilitation).

A total of 4 eMBPs [54,82,87,93] did not have predefined sessions. They were delivered through mobile apps with various exercises and materials from which participants could freely choose according to their own preferences.

More than half of the studies (n=15; Multimedia Appendix 3) included synchronous or asynchronous personal contact with the facilitator via videoconferences, telephone calls, or emails to get regular feedback on their practice in the program.

The intervention duration was usually 8 weeks (n=10), with a session frequency of once per week, ranging from 2 weeks to 6 months. Of all 24 studies, only 2 included a retreat day [62,95].

### Risk of Bias Assessment

The results of the assessment of the risk of bias in the included studies are reported in Multimedia Appendix 3. Of the 24 included studies, 3 did not report all outcomes [75,82,91], 9 had incomplete outcome data (<15% attrition) or did not use ITT analysis [54,71,75,76,78,80,82,85,92], and 10 did not have a comparison group (pre-post studies mentioned earlier). Of the 14 RCT studies, 2 were categorized as unclear regarding random sequence allocation and allocation concealment [85,92] and 2 were categorized as unclear regarding the similarity of groups at baseline or using an appropriate adjustment [87,89]. None of the studies met the criteria for blinding of participants, personnel, or outcome assessment, resulting in a high risk of bias (note that because of the nature of the included studies, the blinding of participants, personnel, and outcome assessment was not possible).

### Feasibility

The reviewed studies examined feasibility in terms of attrition, retention, and adherence. All studies that examined feasibility as a primary outcome (n=12) concluded that eMBPs are feasible for patients with cancer [54,75,78-83,85,87,94,95]. In this review, we summarize the feasibility in terms of attrition and adherence.

#### Attrition

All 24 included studies reported clear data for computing the attrition rate. The attrition rate varied between 6% [79] and 46% [76,82], with an average of 25.3%. These in detail in Multimedia Appendix 3.

##### Predictors of Attrition

Regarding predictors of attrition, 9 studies reported the results of their analysis [54,62,71,73,76,77,80,89,91,93]. Participants who dropped out of the intervention (or did not respond to postassessment queries) had, in comparison with those who did not drop out, lower education [54,62,77], lower income [54], lower relationship satisfaction [76], lower baseline quality of life [77,93], higher baseline pain [93], shorter time since diagnosis [54], brain metastasis [89], worse prognosis [77], less often breast cancer and more often *other* cancer types [77], and more often breast cancer and less often prostate cancer [91]; were more often younger [89], older [91], women [62,91], and men [77]; had comorbidities and were less often occupied by household activities [77]; and participated in an eHealth program rather than an in-person program [62]. However, the authors of 2 studies did not find any differences between completers and noncompleters [71,80]. In addition, the therapeutic alliance did not predict treatment dropout [73].

#### Adherence

There is vast heterogeneity in adherence measures in the reviewed studies. The studies have most often presented these measures of adherence as session completion (including session attendance), practice frequency (eg, how often participants meditated), and practice time (eg, time meditated). The summary is presented in Multimedia Appendix 3.

##### Session Completion

Regarding the session completion of all 24 included studies, only 14 studies could be assessed, 7 studies did not have predefined sessions [54,79,81,82,85,87,93], and 3 studies did not report session completion data [75,88,94].

Of these 14 studies, 6 had comparable results. The other 8 studies [71,76,78,80,83,89,91,92] are limited in terms of this comparison because of considerable heterogeneity in the type of reported participants (enrollers, completers, and unclear) and in the benchmark of minimum completed sessions (50%, 66%, and 100%). In the 6 comparable studies [62,77,84,86,90,95], at least half of the program was completed by 52% [86] to 83% [90] of the enrolled participants (on average across these studies, 70%).

There is a consensus among some authors [8,62] that the minimum adequate *dose attendance* of MBCT is 4 of 8 overall sessions (half of the program). In this context, based on the 6 comparable studies, eMBPs are on average feasible for 70% of participants.

##### Practice Frequency

Of all 24 included studies, 7 reported the practice frequency [54,78,80,82,88,93,94]. In only 2 studies [87,94], it was possible to derive the practice frequency adherence rate. In a study by Kubo et al [87], 50% of completers were adherent for at least 50%, and in a study by Russel et al [94], 61% to 80% of completers were 100% adherent. In the remaining 5 studies [62,78,82,88,93], the adherence rate could not be calculated because the minimum recommended time or the proportion of participants who were adherent was not defined.

##### Practice Time

Of all 24 included studies, 7 reported the practice time [62,78,81,85,91,93,95], but the practice time adherence rate could be derived only in 2 studies. In one study [81], 67% of completers were adherent to 100%, and in another study [85], 56% of completers were adherent to 70% of the recommended time.

In the remaining 5 studies [62,78,91,93,95], the adherence rate could not be calculated because the minimum recommended time or the proportion of participants who were adherent was not defined.

##### Predictors of Nonadherence

Regarding predictors of nonadherence, 5 studies reported the results of their analysis [76,77,82,84,91]. Participants who were nonadherent were more often men [76,77], were more often depressed at baseline [77], had less depressive symptoms [82], had a lower education [77], had a paid job less often [77], had no previous experience with mindfulness [77], and used sleeping medication less often [77]. In 2 studies [77,91], no differences in baseline characteristics were found between adherent and nonadherent participants.

### Efficacy

**Figure 2:**
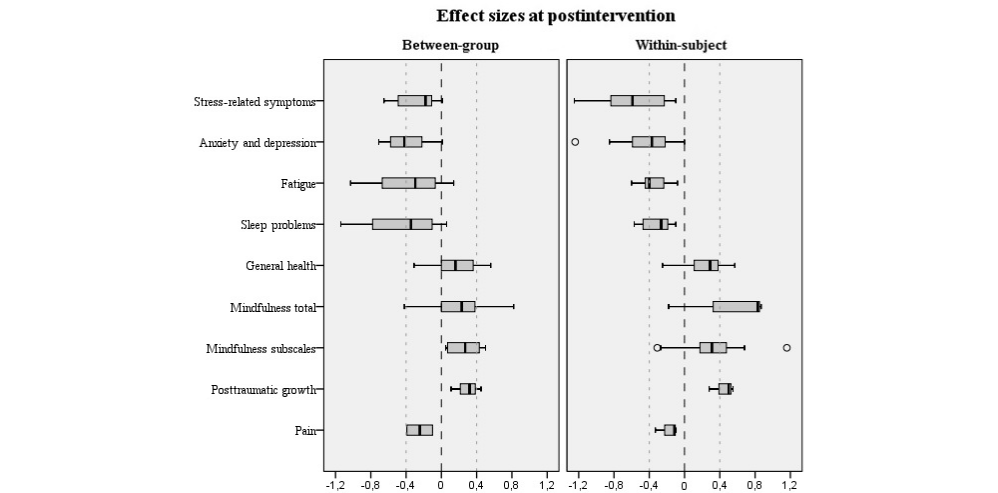
The effect sizes of eHealth mindfulness-based programs on measured outcomes at postintervention.

The efficacy results of eMBPs in patients with cancer on all monitored variables are reported in Multimedia Appendix 3. The main effect of interventions on measured outcomes is presented in Figure 2. In the text of the results section, only the outcomes that were measured by at least four studies are reported; in studies with multiple follow-up endpoints, only the results at the postintervention and the last follow-up endpoint are reported. When multiple measurement instruments were used to measure the same outcome domain within the same time frame, the average effect estimate was calculated. Note that negative effect sizes are indicative of beneficial effects of interventions for outcomes: stress-related symptoms, anxiety and depression, fatigue, sleep problems, and pain.

eMBPs were compared with active controls in 7 studies [62,84-86,89,90,92]. The Study Characteristics section provides the details of the type of active controls. Between-group results of eMBPs compared with no treatment and minimal treatment controls are summarized together, as differences consistently moderated by these control conditions by the phenomenological exploration were not found, and a recent review by Goldberg et al [17] showed that comparing MBPs against no treatment and minimal treatment controls did not differ; Cohen *d*<0.2. The results of comparisons to specific active interventions are reported below.

#### Stress-Related Symptoms

##### Results of Within-Subject Comparisons

The results at postintervention were reported by 8 studies [54,75,76,80,81,87,92,95]; Cohen *d* ranged from −1.25 to −0.10 (median −0.43, IQR −0.84 to −0.23). The results at follow-up were reported by only one study [76], where Cohen *d* of the female subgroup was −0.57 and that of the male subgroup was 0.01.

##### Results of Between-Group Comparisons

The results at postintervention were reported by 5 studies [71,87,91,94,95]; Cohen *d* ranged from −0.65 to 0.01 (median −0.18, IQR −0.49 to −0.11). The results at follow-up were reported by 4 studies [71,86,89,91]; Cohen *d* ranged from −0.68 to 0.05 (median −0.17, IQR −0.39 to −0.02).

#### Anxiety and Depression

Anxiety and depression outcomes are reported together for better summation, as some studies reported both anxiety and depression result in one merged total score [62,70,77,84].

##### Results of Within-Subject Comparisons

The results at postintervention were reported by 10 studies [54,75,76,78,80,81,83,85,87,92]; Cohen *d* ranged from −1.24 to −0.1 (median −0.38, IQR −0.62 to −0.27). The results at follow-up were reported by 2 studies [70,77], with Cohen *d*=−0.71 [77] and *d*=−0.32 [70].

##### Results of Between-Group Comparisons

The results at postintervention were reported by 4 studies [62,72,87,91]; Cohen *d* ranged from −0.71 to 0.01 (median −0.42, IQR −0.58 to −0.22). The results at follow-up were reported by 5 studies [84,86,89-91]; Cohen *d* ranged from −0.53 to 0.14 (median −0.19, IQR −0.37 to −0.06).

#### Fatigue

##### Results of Within-Subject Comparisons

The results at postintervention were reported by 7 studies [54,75,81,83,85,87,92]; Cohen *d* ranged from −0.6 to −0.08 (median −0.40, IQR −0.43 to −0.25). The results at follow-up were reported by 1 study [77], with Cohen *d*=−1.45.

##### Results of Between-Group Comparisons

The results at postintervention were reported by 4 studies [71,85,87,88]; Cohen *d* ranged from −1.03 to 0.14 (median −0.30, IQR −0.49 to −0.18). The results at follow-up were reported by 3 studies [71,84,90]; Cohen *d* ranged from −0.69 to 0.07 (median −0.23, IQR −0.38 to −0.13).

#### Sleep Problems

##### Results of Within-Subject Comparisons

The results at postintervention were reported by 6 studies [54,75,80,81,85,87]; Cohen *d* ranged from −0.57 to −0.1 (median −0.27, IQR −0.44 to −0.19). None of the studies reported results at follow-up.

##### Results of Between-Group Comparisons

The results at postintervention were reported by 4 studies [72,87,88,91], Cohen *d* ranged from −1.14 to 0.06 (median −0.35, IQR −0.60 to −0.19). The results at follow-up were reported by 2 studies, with Cohen *d*=−0.32 [96] and *d*=0.04 [91].

#### General Health

General health includes all measurements assessing physical functioning, mental health, disability, and quality of life.

##### Results of Within-Subject Comparisons

The results at postintervention were reported by 6 studies [54,75,80,81,85,87]; Cohen *d* ranged from −0.25 to 0.57 (median 0.29, IQR 0.11 to 0.38). The results at follow-up were reported by only one study [62], with Cohen *d*=0.44.

##### Results of Between-Group Comparisons

The results at postintervention were reported by 6 studies [62,71,85,87,91,93]; Cohen *d* ranged from −0.31 to 0.56 (median 0.16, IQR 0.02 to 0.33). The results at follow-up were reported by 5 studies [71,86,90,91,93]; Cohen *d* ranged from −0.12 to 0.43 (median 0.10, IQR −0.10 to 0.22).

#### Mindfulness

Some studies reported the results of mindfulness measurements by total score, but others by subscales only (eg, observing, describing, acting with awareness, nonjudging, nonreacting). As these results are incomparable, they are reported separately. First, we report results by total scores, and then we report the results of the subscales only.

##### Results of Within-Subject Total Score Comparisons

The results at postintervention were reported by 3 studies [78,79,81], Cohen *d* ranged from −0.18 to 0.87 (median 0.33, IQR 0.07 to 0.58). Neither of the studies reported results at follow-up.

##### Results of Between-Group Total Score Comparisons

The results at postintervention were reported in 5 studies [62,71,92-94]; Cohen *d* ranged from −0.42 to 0.82 (median 0.23, IQR 0.00 to 0.38). The results at follow-up were reported by 2 studies [71,93], with Cohen *d*=0.00 [71] and *d*=0.3 [93].

##### Results of Within-Subject Subscales Comparisons

The results at postintervention were reported by 4 studies [75,81,87,95]; Cohen *d* ranged from −0.31 to 1.16 (median 0.31, IQR 0.18 to 0.48). None of the studies reported results at follow-up.

##### Results of Between-Group Subscales Comparisons

The results at postintervention were reported by 2 studies [87,95]; Cohen *d* ranged from 0.05 to 0.5 (median 0.27, IQR 0.10 to 0.42). The results at follow-up were reported by only one study [86]; Cohen *d* ranged from 0.03 to 0.16.

#### Posttraumatic Growth

#### Results of Within-Subject Comparisons

The results at postintervention were reported by 3 studies [78,87,95]; Cohen *d* ranged from 0.28 to 0.55 (median 0.42, IQR 0.35 to 0.48). All 3 studies [78,87,95] reported statistically significant improvements. None of the studies reported results at follow-up.

#### Results of Between-Group Comparisons

The results at postintervention were reported by 3 studies [71,87,95]; Cohen *d* ranged from 0.11 to 0.45 (median 0.32, IQR 0.22-0.39). A statistically significant improvement (in favor of the intervention group) was reported by only one study [71]. The results at follow-up were reported by 2 studies [71,86], with Cohen *d*=0.15 [86] and *d*=0.21 [71]. A statistically significant difference (improvement in favor of the intervention group) was reported by 1 study [71].

#### Pain

##### Results of Within-Subject Comparisons

The results at postintervention were reported by 4 studies [81,83,85,87]; Cohen *d* ranged from −0.33 to −0.10 (median −0.12, IQR −0.17 to −0.11). Neither of the studies reported results at follow-up.

##### Results of Between-Group Comparisons

The results at postintervention were reported by 2 studies, with Cohen *d*=−0.39 [87] and *d*=−0.1 [85]. The results at follow-up were reported by 1 study [90], with Cohen *d*=−0.10. The reported difference was not statistically significant.

#### Adverse Effects

Some small adverse effects (Cohen *d*=0.2-0.49) were found in some studies on mindfulness (*d*=−0.42) [94] related to acting with awareness (*d*=−0.31), nonjudging (*d*=−0.27) [75], sex-related distress (*d*=0.26), depression (*d*=0.2) in males [76], satisfaction with sexual life (*d*=−0.22), mental health (*d*=−0.25) [85], and social support (*d*=−0.22) [79].

#### eMBP Versus Specific Active Interventions

eMBPs were compared with specific active interventions by 4 studies, published in 5 papers [62,70,84,89,92].

Compen et al [62] compared eMBCT with parallel, face-to-face MBCT and reported that eMBCT is superior for improving fear of cancer recurrence at postintervention (*d*=−0.21) and for improving anxiety and depression at follow-up (*d*=−0.22 [70]). For other outcomes, including rumination, quality of life, and mindfulness, they found comparable results (*d*<0.2) at both postintervention and follow-up time points.

Bruggeman-Everts et al [84] compared web-based eMBCT tailored for improving chronic cancer–related fatigue with physiotherapist-guided AAF therapy encompassing the use of an accelerometer. They reported that eMBCT is inferior for improving fatigue (*d*=0.37) at follow-up. For other outcomes, including positive and negative effects, anxiety, and depression, they found comparable results (*d*<0.2).

Price-Blackshear et al [92], who compared eMBSR completed only by patients with parallel eMBSR completed by couples (patients together with their partners) at postintervention, reported that eMBSR is inferior for improving anxiety (*d*=−0.27), depression (*d*=0.38), stress-related symptoms (*d*=−0.38), and mindfulness (*d*=−0.63), but surprisingly, eMBSR is superior for improving dyadic adjustment (*d*=0.39) and quality of marriage (*d*=0.29). They found comparable results for fatigue and interpersonal mindfulness (*d*<0.2).

Milbury et al [89] compared a couple-based meditation program with a supportive-expressive group at 3-month follow-up and reported that the meditation program was superior for improving depression (*d*=0.59) and cancer-related stress symptoms (*d*=0.54). They found comparable results for spiritual well-being (*d*<0.2).

### Predictors, Moderators, Mediators, and Working Mechanisms of eMBPs Efficacy

Regarding moderators of eMBP efficacy, 9 studies reported the results of their analysis [62,72,73,75,76,80,87,88,91,97]. Participants who reported greater improvements in at least one of the reported outcomes (stress symptoms, depression and anxiety, spirituality, mindfulness skills, posttraumatic growth, quality of life, sleep problems, and pain) were younger [97], were more adherent [54,80,88], were male [97], were female [76], had prior exposure to meditation [75], participated in tandem with their caregiver [75], and reported higher baseline neuroticism [62], poorer baseline global mental health [72], and early therapeutic alliance [73]. No moderation effect was found for cancer stage [97], type [91], or, in contrast to other studies, adherence [91].

Regarding mediators of eMBP efficacy, 2 studies reported the results of their analysis [70,71]. Mindfulness skills [70,71], acceptance [71], fear of cancer recurrence [70], and rumination [70] were found.

## Discussion

### Principal Findings

This systematic review aims to examine the feasibility and efficacy of eMBPs in improving mental health and well-being in patients with cancer, to describe intervention characteristics and delivery modes of these programs, and to summarize the results of the included studies regarding the moderators, mediators, and predictors of efficacy, adherence, and attrition. Although vast heterogeneity in the intervention and population characteristics was found in the reviewed literature, most of the studies suggested that eHealth is an appropriate way to deliver proven mindfulness effects to patients with cancer. In general, the reviewed studies’ results revealed that eMBPs have the potential to improve various outcomes. Some studies suggest that the effects may be maintained long-term after the end of the intervention. In addition, eMBP is equally effective (at postintervention) as a web-based behavior intervention [62,84] and even more effective (at long-term follow-up visits) as original face-to-face MBCT [70] for psychological distress.

Some significant predictors and moderators of attrition, adherence, and efficacy were found among both the participants and the applications across studies; however, any conclusion would be premature.

Although the results of this review are promising, the small number of RCT studies per outcome, substantial variability across studies, nondifferentiation between primary and secondary outcomes in results summarization, and lack of meta-analysis warrant caution in interpreting and generalizing the observed effects and relationships, as they could be overestimated. The results are heterogeneous across studies and vary between null and large effect sizes. This heterogeneity probably reflects the high variability in the population, intervention, and study characteristics. It also mirrors the fact that the bio-psychosocial complexity of oncological disease–induced distress is enormous, and patients differ in their needs. Existing eMBPs have mostly been trying to convert proven face-to-face mindfulness programs to eHealth mode. They have not yet exploited the full potential of eHealth technology options.

### Attrition Rate

The attrition rate varied between 13% and 48% in the reviewed studies. This corresponds with the data presented in the systematic review of eMBPs in clinical and nonclinical populations, where the attrition rate varied between 7.7% and 52% [44]. The average attrition rate of face-to-face modes of mindfulness-based interventions ranges from 3% to 40% in clinical and nonclinical populations [98] or below 25% in most studies on adults with chronic medical diseases [99]. For comparison, in meaning-centered group psychotherapy for patients with advanced cancer in one study, 28.1% of patients dropped out before the start of the group and 24.5% of the participants dropped out after they began treatment [100]. It seems that the attrition rate of patients with cancer may be higher in eMBPs than in face-to-face programs. This is supported by the results by Compen et al [62], who documented that eHealth MBCT had a higher attrition rate than face-to-face MBCT.

### Adherence

#### Measurement

The number of completed modules was the most prevalent measure of adherence. However, it is often unclear in most studies why certain measures of adherence were chosen over others, as the complete definition was missing in assessed studies. For comparison, Donkin et al [101] assembled a list of methods for measuring adherence to e-therapy (log-ins to the program, module completion, time spent on the internet, completion of a predefined activity or use of an internet tool, posts made, pages viewed, replies to emails, forum visits, self-reported completion of offline activities, and print requests made). In this context, the studies have not yet used available adherence markers, and it seems that many eMBP apps make little use of the current technology options. The majority of the abovementioned methods for measuring adherence to e-therapy can be measured automatically. Unfortunately, programming these app analytical tools is costly and time consuming.

If we use MBSR or MBCT programs as a gold standard in this intervention area, there is some consensus among experts, supported by some experimental data, that 4-week mindfulness programs seem to be efficacious for promoting well-being and stress reduction, and this amount of completion can be considered as a minimum adequate *dose* [8,62,102]. On average, two-thirds of the enrolled participants in the reviewed studies (52%-83%) completed at least half of the sessions. These results are similar to those from a review of web-based mindfulness interventions for people with physical health conditions [51], where the completion rate varied between 60% and 94%.

### Efficacy

In general, the review confirmed the hypothesis that mindfulness delivered via eMBPs is able to induce a desirable change in subjectively assessed levels of stress, anxiety, depression, fatigue, sleep problems, mindfulness, posttraumatic growth, pain, and some parameters of general health. The direction of the found effects on most of the mentioned outcomes is consistent with recent meta-analysis results in face-to-face MBPs [23,70] and in eMBP [41].

The effect sizes were highly heterogeneous between studies, regardless of their methodological quality (Multimedia Appendix 3). The high heterogeneity in the efficacy of various outcomes is consistent with most of the literature related to eMBP [44,51], and it could be partially explained by high variability in the interventions, selected populations, and other study characteristics.

A small adverse effect was described in some studies. From a clinical perspective, the adverse effect on mindfulness [75,94], depression [76], some parameters of mental health [85], and social support [79] should be taken seriously. A recent systematic review of eMBPs in patients with medical conditions found no adverse effect on measured outcomes [60]. Another similar review did not report these data [51]. Although these findings are relatively marginal in our review, they raise a crucial question concerning the individuals for whom eMBP is appropriate and for whom it is not. This question is all the more important because there is usually no control over the mental state of patients, especially in anonymous eMBP, in comparison with face-to-face MBPs. Mindfulness training is paradoxical, and the instruction to focus directly on negative emotions goes against the inherent tendency to avoid unpleasant stimuli. To accept reality, whatever it is, represents the essence of the mindfulness approach. Mindfulness practice supports being in contact with whatever appears in an open, accepting, curious, and nonjudgmental manner. It is easy to imagine that some patients are not ready for this kind of mature emotion regulation strategy, such as acceptance and nonjudgmental openness to experience. After all, some patients experiencing extreme stress associated with cancer diagnosis and treatment involuntarily activate automatic psychological processes as defense mechanisms to reduce the anxiety of the painful emotions related to the illness [103,104]. Thus, mindfulness training can go against this self-protective strategy and, in some individuals, may worsen anxiety and depression and decrease mindfulness.

The selection of suitable patients is addressed in standard face-to-face MBPs in a personal interview before its start. However, there is still no clear consensus for whom participation in an MBP may be contraindicated [105]. In eMBPs, this is addressed in only some studies in the form of a phone call or questionnaire. The majority of studies relied solely on exclusion criteria presented in the input questionnaire at the start of the program. In the context of eMBPs, caution is in order.

A relatively small effect was observed in the context of pain relief, especially given that the pain is prevalent in 50.7% of patients with cancer [106] and that stress and pain reduction is the primary goal of MBSR [107] and depression in MBCT [108]. However, these results follow face-to-face mindfulness-based intervention efficacy. A recent meta‐analysis of RCT studies by Cillessen et al [109] also reported a small mindfulness effect on pain (Hedges *g* ranged between 0.18 and 0.20). More generally, Warth et al [110] reported a small effect of psychological intervention (relaxation, cognitive behavioral therapy, music therapy, mindfulness-based and acceptance-based interventions, and supportive-expressive group therapy) on pain intensity (*d*=−0.29; 95% CI −0.54 to −0.05). For mindfulness-based interventions, it was *d*=0.14. For comparison with a less severe diagnosis such as migraine [111], the effect size of MBSR on pain was medium to large (sensory component 0.79 and affective component *d*=0.81). In the context of eMBP, Ljotsson et al [112] used internet-delivered exposure and mindfulness-based therapy for irritable bowel syndrome. The effect size on pain was medium (*d*=0.64). The lower effect of eMBP on pain could be explained as a result of cancer progression over time in many patients in the program. In this context, disease progression should be monitored.

#### Population and Intervention Characteristics Increasing Efficacy

Although there is some knowledge of what the predictors increasing eMBPs adherence and efficacy are, this review shows that only 9 studies analyzed this. Some predictors were mentioned in the results of one study; some of them have been reported repeatedly. Higher adherence within the eMBP protocol and participation in tandem with a caregiver or partner were associated with greater improvements in measured outcomes. Increased mindfulness was also a repeatedly reported mediator of the eMBP effect [70,71]. This finding is consistent with studies documenting mindfulness as one of the main mediators of its effects across the measured outcomes [113-115]. These results underline the importance of adherence. However, the analysis of adherence predictors was presented in only 5 reviewed studies.

#### Participants Characteristics

The 3 predictors of adherence that are mentioned most often in the eHealth research area are age, gender, and education [116-119]; however, findings seemed mixed in patients with cancer [120]. The reviewed studies support this trend with the prediction of gender by 2 studies [76,77] and education by 1 study [77]. This is consistent with findings from other eHealth interventions [121,122] and broader research on health behaviors, indicating that women are more likely to engage in such interventions than men [123]. For men, higher adherence was found in face-to-face psychotherapy [124], which suggests gender preference in different formats of psychological therapy. The role of age-adherence association was not reported in any of the reviewed studies.

The most comprehensive analysis of moderators and predictors from reviewed studies was by Cillessen et al [70]. The investigation revealed that lower levels of psychological distress, rumination, and neuroticism and a higher level of extraversion and agreeableness at the start of the eMBCT and MBCT program predicted less psychological distress at the 9-month follow-up after both interventions. The program-induced changes in mindfulness skills, fear of cancer recurrence, and rumination during both interventions predicted less psychological distress at follow-up. As Cillessen et al [70] discussed, most of these results are in accordance with previous research. For example, a study by Lengacher et al [125] found that patients with more baseline severity had more severe complaints at follow-up (however, patients with more baseline severity benefit relatively more from MBPs than patients with less severity). Other studies [113-115] identified fear of cancer recurrence, rumination, and mindfulness skills as mediators. However, although the authors discuss explanations of why personality traits predict MBP efficacy, some authors have documented the absence of moderation effects of personality traits. Mixed results in terms of the moderation role of personality traits were reported in a review by Vibe et al [126].

#### Intervention Characteristics

We agree with Keleders et al [127] that the critical question in this area concerns which characteristics of web-based or mobile app interventions related to technology and interaction are linked to better adherence.

We found considerable heterogeneity in the platforms and delivery modes of eMBPs across the studies, which makes it impossible to systematically report which of them are associated with the best efficacy. We share this conclusion with other authors [41,51]. In this context, the comparison of different delivery modes of eMBPs, including face-to-face programs in one arm, is still missing. The exception is the study by Compen et al [62], in which the adherence of patients did not differ between eMBCT and MBCT.

A reminder system using email, text messages, or messages on a smartphone is a unique option of eHealth technology [128,129]. The utility of the reminder system also corresponds to our clinical experience with frequent patient statements of how great it would be if somebody could remind them of essential things from their psychotherapy sessions at the right moment in their daily life before problematic behaviors, thoughts, and emotions appeared. Surprisingly, reminders are not widespread across reviewed studies (Table 1), and the considerable heterogeneity of their type, frequency, and content does not allow any conclusion about the feasibility and efficacy of reminders. Wells et al [130] documented the importance and efficacy of smart messaging that reminded oncology patients in an MBCT program of prescribed between‐session activities. The odds of program completion were 8 times greater for patients using smart messaging than for nonusers. There has not yet been a study comparing intervention arms with and without reminders in the field of eMBPs. The utility of the reminder system is nevertheless apparent.

In the context of the beneficial intervention factors reported by Bruggeman-Everts et al [84] from patient feedback, the results of a qualitative study by Compen et al [131] show that the same elements and others (eg, own time management, individual and home setting, website delivery) were reported by patients as beneficial. On the other hand, when patients mentioned a certain aspect as facilitating (eg, the individual setting, not having to cope with other patients’ stories), they also mentioned it as a barrier (no peer support). This was also the case for timing, the individual nature, the asynchronous nature, the diaries, and the importance of self-discipline [131]. As the authors suggest, this ambiguity emphasizes the importance of offering some flexibility in eMBPs [132], so each participant could choose the intervention features and delivery modes according to their own preferences.

Participation in tandem can partially replace group support and facilitation usually present in face-to-face MBPs. Face-to-face MBPs provide patients with social support from others in the group and the development of a therapeutic alliance with lector. The therapeutic alliance, a common factor in psychotherapy, is thought to be an essential factor in its outcomes [133], and it is crucial in mindfulness programs as well [134]. Bisseling et al [73] found that therapeutic alliances are a significant predictor of eMBP efficacy. Although, we could not make any exact conclusions, as research and discussion are still in their early stages [135]; the social support and therapeutic alliance in eMBPs are a topic of clinical relevance for developers and providers of eHealth interventions. The development of technology provides a new practical tool for clinicians and psychologists to be able to take care of patients who are not physically present. Patients can go through the program in the comfort and safety of their homes, at their right time in the context of their medical restrictions, in an anonymous mode (if they want to and if the program allows it), and they can experience it with the real feeling of some kind of alliance with their doctors and psychologists in the treatment journey. In this mode, the therapeutic alliance is not a dyadic but triadic relationship among the user, the e–mental health program, and the program supporter [135]. Some data in the literature even indicate that a therapeutic alliance with an asynchronous e–mental health program can also be stated [136]. Appointments, homework reminders, assessment, and feedback may also help to develop and foster the therapeutic alliance [137]. eMBPs can reduce social isolation and feelings of loneliness, and participation in tandem with a caregiver or a partner is a promising eMBPs option. A synchronous web-based program, for example, the teleconference, allows patients to be in contact with a lector and with the other program participants. Asynchronous eMBPs can offer participants the opportunity to chat with other participants directly via the app or via social networks or some blogging platforms. In the context of adherence and development of a therapeutic alliance, our own clinical experience has led us to prefer a program that is organized and provided by the concrete hospital or center where patients are treated. It allows them to know who is behind the program and enables them to be in contact while giving them a sense of hospital safety. A combination of personal recommendations from their clinicians or clinical psychologists summarized in handouts and short videos presented in hospital waiting rooms with links to the social media platform and web page of the program can create a field where trust, adherence, and therapeutic alliance can emerge, thus increasing the efficacy of the eMBP programs.

### Risk of Bias

This review included both RCT and pre-post studies. Half of the RCT studies (7/14, 50%) and nearly half of the pre-post studies (4/10, 40%) all have measured biases classified as low. In the pre-post studies, a higher risk of bias could be expected because of the predominant pilot designs.

In the context of RCT studies, high-quality studies are needed to establish the effectiveness of eMBPs. The selected criteria of a 15% attrition cutoff for risk of bias assessments in the current systematic review could seem quite conservative for eHealth studies. The mean attrition rate in the reviewed studies was 25%. The mitigation of this requirement would lower the risk of bias for 1 study [71].

### Limitations

The results of this review have some limitations. First, despite the growing empirical literature on the efficacy of eMBPs in patients with cancer, only a relatively small number of RCT studies have been published, so this review has a relatively small number of RCT studies per outcome.

Second, a summarization of effect estimates was used as a synthesizing method rather than a meta-analysis. This method does not account for differences in the relative sizes of the studies, and the performance of statistics applied in the context of summarizing effect estimates has not been evaluated.

Third, the effectiveness of the included studies varied considerably in terms of outcome, which may be explained by variability in study characteristics, such as participants with different diagnoses and their staging, heterogeneous intervention types (eg, ACT, MBSR, MBCT), various modes of delivery, and outcome measures.

Finally, this review does not differentiate between primary and secondary outcomes to summarize the results. 

The relatively small number of RCT studies per outcome, lack of meta-analysis, substantial variability across studies, and nondifferentiation between primary and secondary outcomes in result summarization warrants caution in interpreting and generalizing the observed effects and relationships.

### Future Directions

We are still at the beginning of answering the questions for whom, with what kind of suffering, in what period of life and disease, and at what readiness to change unhealthy behavior and regulate emotions eMBP is the best tool. We have increasing evidence that eMBPs are useful. However, it must be noted that eMBPs are only one of many other tools in the repertoire of the experienced clinician or clinical psychologist. The effect of eMBP could be maximized when it is recommended to patients suitable for this kind of intervention and in the most effective delivery mode. To answer these questions, we need to provide eMBP to a large sample of patients in properly designed RCT studies in which to manipulate various variables in different study arms (eg, reminders, introductory lectures with the facilitator, internet chat, web, app, or combinations, rewards, etc) in different patient subgroups (eg, by stage of cancer, by readiness to change their behavior and attitudes, with or without psychotherapy). Future research should verify beneficial effects and their moderators found in this exploratory review within RCT studies or within a review incorporating standard meta-analytical methods when more suitable RCT studies will be published.

### Conclusions

To our knowledge, this is the first systematic review that evaluates the feasibility and efficacy of eMBPs in patients with cancer on various psychological and somatic outcomes. The results show that eMBPs are feasible and may be effective in improving various outcomes, especially anxiety and depression and posttraumatic growth. Thus, eHealth represents an appropriate way for mindfulness programs to be delivered to patients with cancer, and they may be even more effective than standard group face-to-face MBPs in some cases. Regarding moderators, a preliminary phenomenological exploration showed possibly important population and intervention factors, such as age, gender, and delivery mode. Although the results of this review are promising, it is still necessary to be wary in interpreting and generalizing the observed effects and relationships.

## Appendix

Multimedia Appendix 1 Search strategy.

Multimedia Appendix 2 Characteristics of included studies (Table 2).

Multimedia Appendix 3 Results of included studies (Table 3) and Risk of bias assessment (Table 4).

## Abbreviations

AAF: ambulant activity feedback
ACT: acceptance and commitment therapy
eMBP: eHealth mindfulness-based program
ITT: intention-to-treat
MBCR: mindfulness-based cancer recovery
MBCT: mindfulness-based cognitive therapy
MBCT-Ca: mindfulness-based cognitive therapy for cancer
MBP: mindfulness-based program
MBSR: mindfulness-based stress reduction
mHealth: mobile health
PRISMA: Preferred Reporting Items for Systematic Reviews and Meta-Analysis
RCT: randomized controlled trial
