# Supplementary material for: Mindfulness-Based Programs for Patients With Cancer via eHealth and Mobile Health: Systematic Review and Synthesis of Quantitative Research
Source: J Med Internet Res. 2020 Nov 16;22(11):e20709. doi: 10.2196/20709 (PMC7704284; doi:10.2196/20709)
Supplement: Multimedia Appendix 1 [file jmir_v22i11e20709_app1.pdf]

Multimedia Appendix 1

Search strategy

All terms used for electronic database search applied in an example of MEDLINE database search strategy:

MEDLINE (via Pubmed)

#1 Mindfulness[Mesh] OR Acceptance and Commitment Therapy[Mesh] OR Meditation[Mesh]

#2 mindfulness[tiab] OR mindful[tiab] OR "Acceptance and Commitment Therapy"[tiab] OR meditation[tiab] OR meditations[tiab] OR vipassana[tiab] OR MBSR[tiab] OR MBCT[tiab] OR MBCR[tiab] OR MBI[tiab] OR MBIs[tiab] OR IBMT[tiab] OR self-compassion[tiab] OR "body-mind"[tiab]

#3 psycho-oncology[mesh] OR cancer survivors[mesh] OR neoplasms[mesh]

#4 oncology[tiab] OR cancer[tiab] OR neoplasms[tiab]

#5 intervention[tiab] OR program[tiab] OR treatment[tiab] OR therapy[tiab] OR psychotherapy[tiab] OR course[tiab] OR interventions[tiab] OR programs[tiab] OR treatments[tiab] OR therapies[tiab] OR psychotherapies[tiab] OR courses[tiab]

#6 self care[mesh] OR electronic mail[mesh] OR internet[mesh] OR telemedicine[mesh] OR videoconferencing[mesh] OR multimedia[mesh] OR mobile applications[mesh] OR smartphone[mesh] OR telephone[mesh] OR cell phone[mesh] OR text messaging[mesh]

#7 self-help[tiab] OR self-care[tiab] OR guided[tiab] OR email[tiab] OR e-mail[tiab] OR emails[tiab] OR e-mails[tiab] OR internet[tiab] OR web[tiab] OR website[tiab] OR online[tiab] OR digital[tiab] OR virtual[tiab] OR eHealth[tiab] OR e-Health[tiab] OR "e-mental health"[tiab] OR etherapy[tiab] OR e-therapy[tiab] OR mhealth[tiab] OR distance[tiab] OR "minimal contact"[tiab] OR remote[tiab] OR telemedicine[tiab] OR telepsychology[tiab] OR telecare[tiab] OR telehealth[tiab] OR teleconferencing[tiab] OR teleconference[tiab] OR teleconferences[tiab] OR videoconference[tiab] OR videoconferencing[tiab] OR videoconferences[tiab] OR multimedia[tiab] OR computer[tiab] OR computerized[tiab] OR mobile[tiab] OR smartphone[tiab] OR smartphones[tiab] OR iphone[tiab] OR iphones[tiab] OR ipad[tiab] OR ipads[tiab] OR app[tiab] OR apps[tiab] OR application[tiab] OR applications[tiab] OR telephone[tiab] OR telephones[tiab] OR phone[tiab] OR phones[tiab] OR message[tiab] OR messages[tiab] OR messaging[tiab] OR chat[tiab] OR reminders[tiab]

#8 #1 OR #2

#9 #3 OR #4

#10 #6 OR #7

#11 #5 AND #8 AND #9 AND #10
