# Supplementary material for: Mindfulness-Based Programs for Patients With Cancer via eHealth and Mobile Health: Systematic Review and Synthesis of Quantitative Research
Source: J Med Internet Res. 2020 Nov 16;22(11):e20709. doi: 10.2196/20709 (PMC7704284; doi:10.2196/20709)
Supplement: Multimedia Appendix 2 [file jmir_v22i11e20709_app2.pdf]

Multimedia Appendix 2

Characteristics of included studies (Table 2)

Table 2. Characteristics of included studies (note that the outcomes of included studies are reported in Multimedia Appendix 3).

Study	Cancer type; cancer stage; primary treatment; country	Mean age (SD) %F[a]	Intervention groups		Program structure; facilitation; delivery modes	Reminders; retreat day; number of sessions (duration); intervention duration
			Intervention type, n (%)	Control condition, n (%)		
Atreya et al. (2018) [75]	Patients with intestinal cancer; stage IV; receiving primary treatment; the United States	52 (N/A) 64%	First-generation MBP[b], 25 (100); eHealth MBSR[c]	—	Predefined; nonfacilitated; emails, booklet, audio recordings, text messages	Daily (text messages); no retreat day; 8 sessions (N/A[d]); 8 weeks
Brotto et al. (2017) [76]	Patients with colorectal and gynecologic cancer; stages not specified; primary treatment completed; the United States and Canada	56 (8.2) 75%	Mindfulness-informed program, 113 (100.0); mindfulness components in sexuality-related psychoeducation	—	Predefined; facilitated; website, audio recordings	Once per week (emails, phone calls); no retreat day; 12 sessions (60min); 12 weeks
Bruggeman-Everts et al. (2017) [84]	A mix of patients with cancer; stages not specified; primary treatment completed; the Netherlands	55 (9.9) 74%	Adapted MBP, 55 (32.9); eHealth MBCT[e]	Ambulant activity feedback accelerometer, 62 (37.1); psychoeducation, 50 (29.9)	Predefined; facilitated; website, audio recordings	Once per week (emails with therapist); no retreat day; 9 sessions (N/A); 9 weeks
Bruggeman-Everts et al. (2015) [77]	A mix of patients with cancer; stages not specified; primary treatment completed; the Netherlands	50 (10.7) 76%	Adapted MBP, 55 (100); eHealth MBCT	—	Predefined; facilitated; website, audio recordings; emails	Once per week (emails with therapist); no retreat day; 9 sessions (N/A); 9 weeks
Campo et al. (2017) [78]	A mix of patients with cancer; stages not specified; primary treatment completed; the United States	27 (2.1) 100%	Adapted MBP, 25 (100); mindful self-compassion	—	Predefined; facilitated; videoconferences, audio recordings, emails	Once per week (emails); no retreat day; 8 sessions (90min); 8 weeks
Compen et al. (2018) [62]; Cillessen et al. (2018) [70]; Bisseling et al. (2019) [73]	A mix of patients with cancer; stages 1-4; receiving/after primary treatment; the Netherlands	52 (10.7) 86%	First-generation MBP, 90 (36.8); eHealth MBCT	Face-to-face MBCT, 77 (31.4); usual care, 78 (31.8)	Predefined; facilitated; website, emails audio recordings	Once per week (emails with therapist); 6,5h retreat day; 8 sessions (N/A); 8 weeks

Study	Cancer type; cancer stage; primary treatment; country	Mean age (SD) %F[a]	Intervention groups		Program structure; facilitation; delivery modes	Reminders; retreat day; number of sessions (duration); intervention duration
			Intervention type, n (%)	Control condition, n (%)		
Donovan et al. (2019) [79]	Unclear types of patients with cancer; stages not specified; postprimary treatment; the United States	19 (3.4) 41%	Mindfulness-informed program, 17 (100); mindfulness app	—	Nonpredefined; nonfacilitated; a mobile app, Facebook group, audio recordings, videos	Daily (facebook posts); no retreat day; 28 sessions (N/A); 4 weeks
Hawkes et al. (2014) [71]; Hawkes et al. (2013) [74]	Patients with colorectal cancer; Duke's stage A to C; receiving/after primary treatment; Australia	66 (10.1) 46%	Mindfulness-informed program, 205 (50.0); multiple health behavior intervention using ACT[f] strategies	Usual care, 205 (50.0)	Predefined; facilitated; telephone, booklet	Frequency not specified (postcards); no retreat day; 11 sessions (N/A); 6 months
Huberty et al. (2019) [85]; Puzia et al. (2020) [72]	Patients with myeloproliferative cancer; stages not specified; receiving of treatment not specified; the United States	58 (12) 81%	Mindfulness-informed program, 128 (67.1)[g]; *Calm* or 10% Happier meditation mobile app	Education, 63 (32.9)[g]	Nonpredefined; nonfacilitated; a mobile app, audio recordings	None; no retreat day; no sessions; 4 weeks
Chambers et al. (2017) [86]	Patients with prostate cancer; metastatic stage; received of primary treatment not specified; Australia	70 (8.6) 0%	First-generation MBP, 94 (49.8); eHealth MBCT	Minimally enhanced usual care, 95 (50.2)	Predefined; facilitated; teleconferences, workbook	Once per week (session teleconferences); no retreat day; 8 sessions (85min); 8 weeks
Kinner et al. (2018) [80]	Patients with ovarian cancer; stages not specified; primary treatment completed; the United States	59 (6.9) 100%	Mindfulness-informed program, 28 (100); eHealth MBSR + ACT + CBSM[h]	—	Predefined; facilitated; videoconferences, website, workbook	Once per week (session videoconferences); no retreat day; 10 sessions (105min); 10 weeks
Kubo et al. (2019) [87]	A mix of patients with cancer; stages 0-4; receiving/after primary treatment; the United States	58 (14.5) 70%	Adapted MBP, 28 (39); Headspace mindfulness app	Waitlist control, 43 (61)	Nonpredefined; nonfacilitated; website or mobile app, audio recordings, videos	Frequency not specified (notifications); no retreat day; no sessions; 8 weeks
Kubo et al. (2018) [54]	A mix of patients with cancer; stages 0-4; receiving primary treatment; the United States	65 (N/A) 68%	Adapted MBP, 54 (100); Headspace mindfulness app	—	Nonpredefined; nonfacilitated; mobile or computer app, videos, audio recordings	Frequency not specified (notifications); no retreat day; no sessions; 8 weeks
Lengacher et al. (2018) [81]	Patients with breast cancer; stages 0-3; primary treatment completed; the United States	57 (9) 100%	Adapted MBP, 15 (100); eHealth MBSR	—	Asynchronous; facilitated; a mobile app, videos, audio recordings, booklet	Once per week (telephone calls); no retreat day; 6 sessions (120min); 6 weeks

Study	Cancer type; cancer stage; primary treatment; country	Mean age (SD) %F[a]	Intervention groups		Program structure; facilitation; delivery modes	Reminders; retreat day; number of sessions (duration); intervention duration
			Intervention type, n (%)	Control condition, n (%)		
Messer et al. (2019) [88]	A mix of patients with cancer; stages 1-3; primary treatment completed; the United States	51 (N/A) 80%	Adapted MBP, 11 (52); eHealth MBSR	Usual care, 10 (48)	Predefined; nonfacilitated; website, audio recordings, written materials	None; no retreat day; 6 sessions (N/A); 6 weeks
Mikolasek et al. (2018) [82]	A mix of patients with cancer; stages 0-4; receiving/after primary treatment; the United States	53 (11.5) 74%	Mindfulness-informed program, 100 (100); meditation and relaxation mobile app	—	Nonpredefined; nonfacilitated; a mobile app, audio recordings	Daily (notifications); no retreat day; no sessions; 10 weeks
Milbury et al. (2020) [89]	Patients with lung cancer; metastatic stage; receiving of treatment not specified; the United States	65 (10.4) 51%	Mindfulness-informed program, 26 (35); couple-based meditation program	Usual care, 25 (33); supportive-expressive group, 24 (32)	Predefined; facilitated; videoconferences	Once per week (session videoconferences); no retreat day; 4 sessions (60min); 4 weeks
Mosher et al. (2018) [90]	Patients with breast cancer; metastatic stage; receiving of treatment not specified; the United States	56 (11.4) 100%	Adapted MBP, 23 (49); eHealth ACT	Education, 24 (51)	Predefined; facilitated; telephone calls, audio recordings	Once per week (session calls); no retreat day; 6 sessions (55min); 6 weeks
Nissen et al. (2020) [91]	Survivors of breast cancer and prostate cancer; stages not specified; postprimary treatment; Denmark	54 (N/A) N/A %	First-generation MBP, 104 (69.3); eHealth MBCT	Waitlist control, 46 (30.7)	Predefined; facilitated; website, videos, audio recordings, written materials	Once per week (therapist emails); no retreat day; 8 sessions (120min); 8 weeks
Plumb Vilardaga et al. (2020) [83]	A mix of patients with cancer; stages 3b-4; receiving of treatment not specified; the United States	66 (10.8) 71%	Mindfulness-informed program, 20 (100); coping skills training and ACT	—	Predefined; facilitated; telephone calls, workbook, audio recordings	Once per week (session calls); no retreat day; 4 sessions (45 min); 4 weeks
Price-Blackshear et al. (2020) [92]	Patients with breast cancer; stages 0-3; receiving of treatment not specified; the United States	40 (5.3) 100%	First-generation MBP, 42 (53); eHealth MBSR – individual	Pure MBP, 38 (47); adapted MBSR for couples	Predefined; facilitated; video, audio recordings; emails	Two per week (email reminders); none; 8 sessions (60min); 8 weeks

Study	Cancer type; cancer stage; primary treatment; country	Mean age (SD) %F[a]	Intervention groups		Program structure; facilitation; delivery modes	Reminders; retreat day; number of sessions (duration); intervention duration
			Intervention type, n (%)	Control condition, n (%)		
Rosen et al. (2018) [93]	Patients with breast cancer; Stages 0-4; receiving/after primary treatment; the United States	52 (10.3) 100%	Adapted MBP, 57 (50.9); Headspace mindfulness app	Waitlist control, 55 (49.1)	Nonpredefined; nonfacilitated; a mobile app, videos, audio recordings	Once per week (emails); no retreat day; no sessions; 8 weeks
Russell et al. (2019) [94]	Patients with melanoma cancer; Stages 2c-3; primary treatment completed; Australia	53 (13.7) 54%	Adapted MBP, 46 (67); eHealth MBSR	Usual care, 23 (33)	Predefined; nonfacilitated; website, videos, audio recordings	Twice daily (emails); no retreat day; 6 sessions (N/A); 6 weeks
Zernicke et al. (2014) [95]; Zernicke et al. (2016) [97]	A mix of patients with cancer; stages 1-4; primary treatment completed; Canada	58 (21.2) 73%	Adapted MBP, 30 (48); eHealth MBCR[i]	Waitlist control, 32 (52)	Predefined; facilitated; videoconferences, videos, audio recordings	Once per week (session videoconferences); 6h retreat day; 8 sessions (120min); 8 weeks

[a] Mean age and F% (proportion of female) of the total study population at baseline.

[b] MBP: mindfulness-based programs

[c] MBSR: mindfulness-based stress reduction

[d] N/A: not available

[e] MBCT: mindfulness-based cognitive therapy

[f] ACT: acceptance and commitment therapy

[g] total number of patients that were enrolled in at least one intervention and education control group

[h] CBSM: cognitive-behavioral stress management

[i] MBCR: mindfulness-based cancer recovery
