# Supplementary material for: Mindfulness-Based Programs for Patients With Cancer via eHealth and Mobile Health: Systematic Review and Synthesis of Quantitative Research
Source: J Med Internet Res. 2020 Nov 16;22(11):e20709. doi: 10.2196/20709 (PMC7704284; doi:10.2196/20709)
Supplement: Multimedia Appendix 3 [file jmir_v22i11e20709_app3.pdf]

Multimedia Appendix 3

Results of included studies (Table 3) and Risk of bias assessment (Table 4)

Table 3. Results of included studies

Study	Attrition rate	Adherence sessions completion	Adherence rate practice frequency	Adherence rate practice time	Outcomes[a]	Postintervention [b] Cohen's d		Follow-up[c] Cohen's d	
						Within-subject	Between-group	Within-subject	Between-group
Atreya et al. (2018) [75]	32%	N/A	N/A	N/A		8 weeks[d]			
					Distress (thermometer)	-0.17	—	—	—
					anxiety (PROMIS)	-0.10	—	—	—
					depression (PROMIS)	0.07	—	—	—
					fatigue (PROMIS)	-0.42	—	—	—
					sleep disturbances (PROMIS)	-0.19	—	—	—
					global mental health (PROMIS)	0.11	—	—	—
					global physical health (PROMIS)	N/A	—	—	—
					acting with awareness (FFMQ)	-0.31	—	—	—
					describing (FFMQ)	0.13	—	—	—
					nonjudging (FFMQ)	-0.27	—	—	—
					nonreactivity (FFMQ)	0.31	—	—	—
					observing (FFMQ)	0.20	—	—	—
Brotto et al. (2017) [76]	46%	43% of enrolled participants completed full intervention	N/A	N/A		12 weeks		6 months	
					Sex-related distress F (FSDS)	-0.47	—	-0.57	—
					sex-related distress Ma (FSDS)	0.26	—	0.01	—
					Sexual functioning F (FSFI)	1.04	—	N/A	—
					Sexual functioning Ma (IIEF)	0.28	—	N/A	—
					depression F (BDI)	-0.38	—	N/A	—
					depression Ma (BDI)	0.20	—	N/A	—
Bruggeman-Everts et al. (2017) [84]	27%	62% of enrolled participants attended two thirds or more sessions	N/A	N/A					6 months
					Fatigue severity (CIS-FS)	—	—	—	-0.69
					positive affects (PANAS)	—	—	—	0.04
					negative affects (PANAS)	—	—	—	-0.07
					anxiety and depression (HADS)	—	—	—	-0.04
Bruggeman-Everts et al. (2015) [77]	38%	62% of enrolled participants completed at least two thirds or more of intervention	N/A	N/A				13 weeks	
					Fatigue severity (CIS-FS)	—	—	-1.45	—
					anxiety and depression (HADS)	—	—	-0.71	—

Study	Attrition rate	Adherence sessions completion	Adherence rate practice frequency	Adherence rate practice time	Outcomes[a]	Postintervention [b] Cohen's d		Follow-up[c] Cohen's d	
						Within-subject	Between-group	Within-subject	Between-group
Campo et al. (2017) [78]	29%	84%[e] attended at least 6 out of 8 sessions	Unclear [e f g]; M of formal practice was 3.11 days/week (SD = 1.61); data of 46% enrolled participants N/A	Unclear [e f g]; M = 33.45min/week (SD = 16.59) (= 4.8min/day)		8weeks			
					Anxiety (PROMIS)	-1.24***	—	—	—
					depression (PROMIS)	-0.99***	—	—	—
					social isolation (PROMIS)	-1.10***	—	—	—
					mindfulness (MAAS)	0.87***	—	—	—
					posttraumatic growth (PTGI)	0.50**	—	—	—
					body image (BIS)	-1.39***	—	—	—
					resilience (BRS)	0.33	—	—	—
					self-compassion (SCS)	1.23	—	—	—
Compen et al. (2018) [62]; Cillessen et al. (2018) [70]; Bisseling et al. (2019) [73]	30%	79% of enrolled participants attended half or more sessions	N/A	Unclear[e]; M = 28.7min/day (SD = 29.3min) (R = 30min/day); data of 23% enrolled participants N/A			8weeks	9 months[f g]	
					Anxiety and depression (HADS)	—	-0.71	-0.32	—
					fear of recurrence (FCRI)	—	-0.53	-0.10	—
					rumination (RRQ)	—	-0.51	-0.28	—
					QoL-mental (SF-12)	—	0.67	0.39	—
					QoL-physical (SF-12)	—	0.24	0.00	—
					mindfulness (FFMQ-SF)	—	0.82	N/A	—
					positive mental health (MHC-SF)	—	0.44	0.48	—
Donovan et al. (2019) [79]	6%	On average, completers completed a mean 16.9 (SD 11.9, range 0-28) of the 28 unique sessions	N/A	N/A		4 weeks			
					Social support (PSS-Fr)	-0.22	—	—	—
					mindfulness (CAMM)	-0.18	—	—	—
					psychological functioning (PCQL)	0.14	—	—	—
					body image distress (BIS)	-0.31	—	—	—
Hawkes et al. (2014) [71]; Hawkes et al. (2013) [74]	23%	81% [f h] attended half or more of sessions	N/A	N/A			6 months		12 months
					Mindfulness (MAAS)	—	0.00	—	0.00
					quality of life (FACT-C)	—	0.12	—	0.10
					QoL-physical (SF-36)	—	0.00	—	0.13
					QoL-mental (SF-36)	—	0.07	—	-0.08
					distress (BSI-18)	—	0.01	—	-0.04
					posttraumatic growth (PTGI)	—	0.32	—	0.21
					spirituality (FACIT-sp)	—	0.36	—	0.13
					acceptance (AAQ-II)	—	0.47	—	-0.01
					cancer fatigue (FACIT-FS)	—	0.14	—	0.07
Huberty et al. (2019) [85]; Puzia et al. (2020) [72]	27%	—	N/A	56% of completers was adherent to at least 70%; M = 56% practiced at least 49min/week R = 10min/day data of 27% enrollers are N/A		4 weeks[i]	4 weeks[i]		
					Pain intensity (PROMIS)	-0.11	-0.10	—	—
					anxiety (PROMIS)	-0.22	-0.70[j]	—	—
					depression (PROMIS)	-0.29	-0.04[j]	—	—
					sleep disturbance (PROMIS)	-0.47	-0.42[j]	—	—
					satisfaction w/ sex life	-0.22	-0.21	—	—
					physical health (PROMIS)	0.44	-0.31	—	—
					mental health (PROMIS)	-0.25	-0.26	—	—
					total symptom burden (MPN-SAF)	-0.27	-0.29	—	—
					fatigue (MPN-SAF)	-0.27	-0.31	—	—

Study	Attrition rate	Adherence sessions completion	Adherence rate practice frequency	Adherence rate practice time	Outcomes[a]	Postintervention [b] Cohen's d		Follow-up[c] Cohen's d	
						Within-subject	Between-group	Within-subject	Between-group
Chambers et al. (2017) [86]	22%	52% of enrolled participants attended half or more of sessions	N/A	N/A					9 months
					Psychological distress (BSI-18)	—	—	—	0.19
					cancer-specific distress (IES)	—	—	—	-0.09
					anxiety (MAX-PC)	—	—	—	0.14
					quality of life (FACT-p)	—	—	—	-0.12
					benefit finding (PTGI)	—	—	—	0.15
					observing (FFMQ)	—	—	—	0.11
					describing (FFMQ)	—	—	—	0.07
					acting with awareness (FFMQ)	—	—	—	0.16
					nonjudging (FFMQ)	—	—	—	0.16
					nonreactivity (FFMQ)	—	—	—	0.03
Kinner et al. (2018) [80]	32%	Overall attendance of completers was 88.9% (169/190 sessions)	Unclear[k]; average of completers was 2.8 times per week (range 0.22-7.33). Data of 32% enrolled participants N/A.	N/A		10 weeks			
					Quality of life (FACT-O)	0.29	—	—	—
					perceived stress (PSS)	-0.43	—	—	—
					mood disturbances (POMS)	-0.28	—	—	—
					depression (CESD)	-0.28	—	—	—
					sleep problems (PSQI)	-0.10	—	—	—
					social support (SPS)	0.12	—	—	—
Kubo et al. (2019) [87]	17%	—	50% of completers were adherent for least 50%; they practiced at least 50% of days (recommendation was to practice every day). Data of 17% enrolled participants N/A.	N/A		8 weeks	8 weeks		
					Distress (thermometer)	-0.29	-0.11	—	—
					anxiety (HADS-anx)	-0.31	-0.40	—	—
					depression (HADS-dep)	-0.37	0.01	—	—
					pain intensity (PROMIS)	-0.35	-0.42	—	—
					pain interference (PROMIS)	-0.31	-0.36	—	—
					sleep disturbance (PROMIS)	-0.19	-0.27	—	—
					fatigue (BFI)	-0.20	-0.28	—	—
					quality of life (FACT-G)	0.29	0.53	—	—
					posttraumatic growth (PTGI)	0.28	0.45	—	—
					Observing (FFMQ-SF)	0.24	0.22	—	—
					Describing (FFMQ-SF)	0.15	0.18	—	—
					Act with awareness (FFMQ-SF)	0.36	0.43	—	—
					Nonjudging (FFMQ-SF)	0.24	0.37	—	—
					Nonreacting (FFMQ-SF)	0.48	0.45	—	—
Kubo et al. (2018) [54]	32%	—	N/A	N/A		8 weeks			
					Distress (thermometer)	-1.25	—	—	—
					anxiety (HADS-anx)	-0.79	—	—	—
					depression (HADS-dep)	-0.53	—	—	—
					QoL-physical (PROMIS)	0.38	—	—	—
					QoL-mental (PROMIS)	0.57	—	—	—
					fatigue (BFI)	-0.38	—	—	—
					sleep problems (PSQI)	-0.57	—	—	—

Study	Attrition rate	Adherence sessions completion	Adherence rate practice frequency	Adherence rate practice time	Outcomes[a]	Postintervention [b] Cohen's d		Follow-up[c] Cohen's d	
						Within-subject	Between-group	Within-subject	Between-group
Lengacher et al. (2018) [81]	13%	—	N/A	67% of completers was adherent to 100%; average of all participants was 36min/day and 67% of them practiced higher than 20min/day as recommended; data of 20% enrolled participants N/A		6 weeks			
					QoL-physical (SF-36)	0.29	—	—	—
					QoL-mental (SF-36)	0.11	—	—	—
					observing (FFMQ)	0.64	—	—	—
					describing (FFMQ)	0.15	—	—	—
					acting with awareness (FFMQ)	0.53	—	—	—
					nonjudging (FFMQ)	1.16	—	—	—
					nonreactivity (FFMQ)	0.68	—	—	—
					mindfulness total (FFMQ)	0.83	—	—	—
					cognitive impairment (EoCG)	-0.66	—	—	—
					depression (CESD)	-0.85	—	—	—
					anxiety (STAI-S)	-0.72	—	—	—
					perceived stress (PSS)	-0.84	—	—	—
					fear of recurrence (CARS)	-0.74	—	—	—
					sleep problems (PSQI)	-0.34	—	—	—
					fatigue symptoms (FSI)	-0.60	—	—	—
					fatigue interference (FSI)	-0.47	—	—	—
					pain severity (BPI)	-0.03	—	—	—
					pain interference (BPI)	-0.16	—	—	—
Messer et al. (2019) [88]	9%	N/A	Unclear[e]; M = 13 exercises over the course (approx. 2 exercises/week) R: daily data of 9% enrolled participants N/A	N/A			6 weeks[l]		
					Depression and anxiety (HADS)	—	-0.64	—	—
					mood disturbance (POMS)	—	-0.67	—	—
					sleep problems (PSQI)	—	-1.14	—	—
					fatigue (FSI)	—	-1.03	—	—
Mikolasek et al. (2018) [82]	46%	—	Unclear[e]; Mdn = 4 (IQR = 1-7; at week 1) and 2 (IQR = 1-4; at week 10) exercises/week of recommended 5 exercises/week; data of 46% enrolled participants N/A	N/A		10 weeks[m]		20 weeks[m]	
					Distress (thermometer)	N/A	—	N/A	—
					anxiety and depression (HADS)	N/A	—	N/A	—
					quality of life (FACT-G)	N/A	—	N/A	—
					health-related QoL (PROMIS)	N/A	—	N/A	—
					fear of progression (FOP-Q-SF)	N/A	—	N/A	—
					mindfulness (FMI)	N/A	—	N/A	—
Milbury et al. (2020) [89]	27%	76% of participants attended more than half of intervention (at least 3 sessions)	N/A	N/A			4 weeks[n]		3 months[n]
					Depression (CES-D)	—	N/A	—	-0.53
					cancer-related stress (IES)	—	N/A	—	-0.68
					spiritual well-being (FACT-SP)	—	N/A	—	0.26

Study	Attrition rate	Adherence sessions completion	Adherence rate practice frequency	Adherence rate practice time	Outcomes[a]	Postintervention [b] Cohen's d		Follow-up[c] Cohen's d	
						Within-subject	Between-group	Within-subject	Between-group
Mosher et al. (2018) [90]	22%	83% of enrolled participants attended half or more of sessions	N/A	N/A					12 weeks
					Symptom interference (MDASI)	—	—	—	-0.34
					pain interference (PROMIS)	—	—	—	-0.02
					fatigue interference (FSI)	—	—	—	-0.27
					sleep impairment (PROMIS)	—	—	—	-0.38
					pain intensity (PROMIS)	—	—	—	-0.17
					fatigue (PROMIS)	—	—	—	-0.19
					sleep disturbances (PROMIS)	—	—	—	-0.25
					depressive symptoms (PROMIS)	—	—	—	0.01
					anxiety (PROMIS)	—	—	—	-0.10
Nissen et al. (2020) [91]	26%	56% of enrolled participants completed all eight sessions<br><br>all enrolled participants completed an average of 5.6 sessions out of eight	N/A	Unclear[k o]; M = login time was 6 hours and 38 minutes per completed module			8 weeks		6 months
					Anxiety (STAI-Y)	—	-0.45	—	-0.40
					depression (BDI-II)	—	-0.42	—	-0.28
					stress (PSS)	—	-0.18	—	-0.29
					mental well-being (WHO-5)	—	0.20	—	0.43
					sleep disturbances (ISI)	—	0.06	—	0.04
Plumb Vilardaga et al. (2020) [83]	12%	95% of enrolled participants completed all four sessions	N/A	N/A		4 weeks[l]			
					Pain severity (BPI-severity)	-0.09	—	—	—
					pain disability (PDI)	-0.15	—	—	—
					fatigue (PROMIS)	-0.08	—	—	—
					anxiety (HADS-anx)	-0.13	—	—	—
					depression (HADS-dep)	-0.17	—	—	—
					acceptance and action (AAQ-II)	-0.12	—	—	—
					values summary (VAL-sum)	-0.17	—	—	—
Price-Blackshear et al. (2020) [92]	26%	77% of completers reported watching all eight assigned session videos	N/A	N/A		8 weeks[p]			
					Anxiety (PROMIS)	-0.59	—	—	—
					depression (PROMIS)	-0.39	—	—	—
					stress (PSS)	-0.75	—	—	—
					fatigue (N/A)	-0.42	—	—	—
					mindfulness (MAAS)	0.23	—	—	—
					dyadic adjustment (DAS)	0.11	—	—	—
					quality of marriage (QMI)	0.14	—	—	—
					interpersonal mindfulness (IMS)	0.39	—	—	—

Study	Attrition rate	Adherence sessions completion	Adherence rate practice frequency	Adherence rate practice time	Outcomes[a]	Postintervention [b] Cohen's d		Follow-up[c] Cohen's d	
						Within-subject	Between-group	Within-subject	Between-group
Rosen et al. (2018) [93]	46%	—	Unclear[g k]; participants logged into the app on average 18 days over the course (range 1-78 days) (= 2.25 day/week); data of 40% started participants N/A	Unclear [g k]; average duration of app engagement was 13.40 minutes per day (range 10 to 40 minutes). Individual mindfulness sessions ranged 3 to 20 minutes; data of 40% started participants N/A			9 weeks		13 weeks
					General well-being (FACT-B)	—	0.36	—	0.31
					mindfulness (MAAS)	—	0.38	—	0.30
Russell et al. (2019) [94]	30%	N/A	61%-80% of completers were 100% adherent; 61% (at week 2) and 80% (at week 4) practiced every day as recommended; data of 50% (at week 2) and 67% (at week 4) enrolled participants N/A	N/A			6 weeks		
					Fear of cancer recurrence (FCRI)	—	-1.42	—	—
					rumination (RRQ-rum)	—	-1.49	—	—
					mindfulness (CAMS-R)	—	–0.42	—	—
					worries (PSQW-A)	—	–0.34	—	—
					stress (PSS-10)	—	-0.65	—	—
Zernicke et al. (2014) [95]; Zernicke et al. (2016) [97]	18%[i]	82% of enrolled participants completed half or more sessions[g]	N/A	Unclear[g k] M = 134min/5days week (= 26.8min/day) (R = 45min/day); data of 18% enrolled participants N/A		8 weeks[r]	8 weeks[r]		
					Mood disturbancess (POMS)	-0.61	-0.44	—	—
					stress (CSOSI)	-0.83	-0.49	—	—
					spiritual well-being (FACIT-sp)	0.60	0.37	—	—
					posttraumatic growth (PTGI)	0.55	0.11	—	—
					observing (FFMQ)	0.31	0.05	—	—
					describing (FFMQ)	0.32	0.06	—	—
					acting with awareness (FFMQ)	0.47	0.50	—	—
					nonjudging (FFMQ)	0.45	0.32	—	—
					nonreactivity (FFMQ)	0.57	0.07	—	—

Abbreviations: AAQ-II, Acceptance and Action Questionnaire; BDI, Beck Depression Inventory; BFI, Brief Fatigue Inventory; BIS, Body Image Scale; BPI, Brief Pain Inventory; BRS, Brief Resilience Scale; BSI-18, Brief Symptom Inventory; BPI-severity, Brief Pain Inventory – Pain Severity Subscale; CAMM, Child and Adolescent Mindfulness Measure; CAMS-R, Cognitive and Affective Mindfulness Scale–Revised; CARS, Concerns About Recurrence Scale; CESD, Center for Epidemiologic Studies Depression; CIS-FS, Checklist Individual Strength—Fatigue Severity; CSOSI, Calgary Symptoms of Stress Inventory; DAS, Dyadic Adjustment Scale; ECog, Everyday Cognition; ERQ, Emotion Regulation Questionnaire; ES, effect size/s; F, female; FACIT(-FS; -SP), Functional Assessment of Chronic Illness Therapy (—Fatigue Scale; —Spirituality); FACT(-B; -C; -G; -P; -O) Functional Assessment of Cancer Therapy (—Breast; —Colorectal; —General; —Prostate; —Ovarian); FCRI, Fear of Cancer Recurrence Inventory; FMI , Freiburg Mindfulness Inventory; FFMQ (-SF), Five Facets of Mindfulness Questionnaire (—Short Form); FOP-Q-SF, Fear Of Progression Questionnaire—Short Form; FSDS, Female Sexual Distress Scale; FSFI, Female Sexual Function Index, FSI, Fatigue Symptom Inventory; HADS, Hospital Anxiety and Depression Scale; IES, Impact of Event Scale; IIEF, International Index of Erectile Function; IMS, Interpersonal Mindfulness Scale; ISI, Insomnia Severity Index; Ma, male; MAAS, Mindful Attention Awareness Scale; MAX-PC, Memorial Anxiety Scale for Prostate Cancer; MDASI, MD Anderson Symptom Inventory; MHC-SF, Mental Health Continuum—Short Form M, mean; Mdn, median; mo, month/s; MPN-SAF, MPN Symptom Assessment Form; N/A, data not available; PANAS, Positive and Negative Affect Schedule; PCQL-32-Pf, Pediatric Cancer Quality of Life Inventory 32-Psychological Functioning subscale; PDI, Pain Disability Index; PSQI, Pittsburgh Sleep Quality Index; PROMIS, Patient–Reported Outcomes Measurement Information System; PSWQ-A, Penn State Worry Questionnaire—Abbreviated; PSS, The Perceived Stress Scale; PSS-Fr,

Social Support, Friends; POMS, Profile of Mood States PTGI, Posttraumatic Growth Inventory; QoL, quality of life; QMI, Quality of Marriage Index; R, recommendation; RRQ, Rumination and Reflection Questionnaire; SCS, Self-Compassion Scale; SF-12/SF-36, Short Form Health Survey; SPS, Social Provisions Scale; STAI-S, State scale of State Trait Anxiety Inventory; VAL-sum, Bull´s Eye Values Inventory – Summary score; we, weeks; WHO-5, World Health Organisation 5-item well-being index;

[a] primary outcomes are initialized

[b] baseline to postintervention assessment data (duration is reported above the effect sizes)

[c] baseline to follow up assessment data (duration is reported above the effect sizes)

[d] data of unpaired patients only not applicable

[e] unclear what proportion of participants were adherent to what proportion of recommended practice

[f] practice frequency/duration recommendation is unclear

[g] unclear how many participants were not reported

[h] unclear if of those who were enrolled or who completed the intervention

[i] only the results of *Calm* app are reported as it was superior to *10% Happier* app in terms of feasibility and authors recommend it for future trials

[j] results of participants who used *Calm* app (29) during the first 4 weeks of the study (before having used another app or received education) compared to those who received an education control (51) also during the first 4 weeks

[k] postintervention to follow-up assessment data

[l] in this case, authors reported effect size as Hedge´s g_{av}

[m] results of assessed psychosocial outcomes was not reported

[n] results of mindfulness group compared to usual care group are reported here

[o] data of sample expanded by waitlist group which completed the intervention afterward

[p] only the results of individual Mindfulness-based intervention are reported

[r] data of original intervention sample (not of expanded by waitlist group which completed the intervention afterward)

Table 4. Risk of bias assessment

Study	Selection bias		Performance and detection bias		Attrition bias	Reporting bias	Other biases	
	1. Random sequence generation	2. Allocation concealment	3. Blinding of participants and personnel	4. Blinding of outcome assessment	5. Complete outcome data or intention-to-treat analysis used	6. All outcomes reported	7. Similar groups	8. Presence of control group
Atreya et al. (2018) [75]	—	—	—	—	No	No	—	No
Brotto et al. (2016) [76]	—	—	—	—	No	No	—	No
Bruggeman-Everts et al. (2017) [84]	Yes	Yes	No	No	Yes	Yes	Yes	Yes
Bruggeman-Everts et al. (2015) [77]	—	—	—	—	Yes	Yes	—	No
Campo et al. (2017) [78]	—	—	—	—	No	Yes	—	No
Compen et al. (2018) [62]; Cillessen et al. (2018) [70]; Bisseling et al. (2019) [73]	Yes	Yes	No	No	Yes	No	Yes	Yes
Donovan et al. (2019) [79]	—	—	—	—	Yes	Yes	—	No
Hawkes et al. (2014) [71]; Hawkes et al. (2013) [74]	Yes	Yes	No	No	No	Yes	Yes	Yes
Huberty et al. (2019) [85]; Puzia et al. (2020) [72]	Unclear	Unclear	No	No	No	Yes	Yes	Yes
Chambers et al. (2017) [86]	Yes	Yes	No	No	Yes	Yes	Yes	Yes
Kinner et al. (2018) [80]	—	—	—	—	No	Yes	—	No
Kubo et al. (2019) [87]	Yes	Yes	No	No	Yes	Yes	Unclear	Yes

Study	Selection bias		Performance and detection bias		Attrition bias	Reporting bias	Other biases	
	1. Random sequence generation	2. Allocation concealment	3. Blinding of participants and personnel	4. Blinding of outcome assessment	5. Complete outcome data or intention-to-treat analysis used	6. All outcomes reported	7. Similar groups	8. Presence of control group
Kubo et al. (2018) [54]	—	—	—	—	No	Yes	—	No
Lengacher et al. (2018) [81]	—	—	—	—	Yes	Yes	—	No
Messer et al. (2019) [88]	Yes	Yes	No	No	Yes	Yes	Yes	Yes
Mikolasek et al. (2018) [82]	—	—	—	—	No	No	—	No
Milbury et al. (2019) [89]	Yes	Yes	No	No	Yes	Yes	Unclear	Yes
Mosher et al. (2018) [90]	Yes	Yes	No	No	Yes	Yes	Yes	Yes
Nissen et al. (2020) [91]	Yes	Yes	No	No	Yes	No	Yes	Yes
Plumb Vilardaga et al. (2020) [83]	—	—	—	—	Yes	Yes	Yes	No
Price-Blackshear et al. (2020) [92]	Unclear	Unclear	No	No	No	Yes	Yes	Yes
Rosen et al. (2018) [93]	Yes	Yes	No	No	Yes	Yes	Yes	Yes
Russell et al. (2019) [94]	Yes	Yes	No	No	Yes	Yes	Yes	Yes
Zernicke et al. (2014) [95]; Zernicke et al. (2016) [97]	Yes	Yes	No	No	Yes	Yes	Yes	Yes
